# Activation of the Coagulation Cascade as a Universal Danger Sign

**DOI:** 10.3390/cimb47020108

**Published:** 2025-02-09

**Authors:** Eleonora A. Starikova, Jennet T. Mammedova, Artem A. Rubinstein, Alexey V. Sokolov, Igor V. Kudryavtsev

**Affiliations:** 1 Laboratory of Cellular Immunology, Department of Immunology, Institute of Experimental Medicine, Akademika Pavlova 12, 197376 Saint Petersburg, Russiaigorek1981@yandex.ru (I.V.K.); 2 Medical Faculty, First Saint Petersburg State I. Pavlov Medical University, L’va Tolstogo St. 6-8, 197022 Saint Petersburg, Russia; 3 Department of Microbiology and Virology, Institute of Medical Education Almazov National Medical Research Centre, 2 Akkuratova Street, 197341 Saint Petersburg, Russia; 4 Department of Molecular Biotechnology, Chemical and Biotechnology Faculty, Saint Petersburg State Institute of Technology, Moskovski Ave., 26, 190013 Saint Petersburg, Russia; 5 Laboratory of Systemic Virology, Department of Molecular Biology of Viruses, Smorodintsev Research Institute of Influenza, 15/17, Prof. Popova Str., 197376 Saint Petersburg, Russia; biochemsokolov@gmail.com

**Keywords:** coagulation and immunity, factor XII, thrombin, fibrinogen, PAMPs, DAMPs, PRRs, uPAR, PAR, inflammation

## Abstract

Hemostasis is a mechanism that stops bleeding from an injured vessel, involves multiple interlinked steps, culminating in the formation of a “clot” sealing the damaged area. Moreover, it has long been recognized that inflammation also provokes the activation of the coagulation system. However, there has been an increasing amount of evidence revealing the immune function of the hemostasis system. This review collects and analyzes the results of the experimental studies and data from clinical observations confirming the inflammatory function of hemostasis. Here, we summarize the latest knowledge of the pathways in immune system activation under the influence of coagulation factors. The data analyzed allow us to consider the components of hemostasis as receptors recognizing «foreign» or damaged «self» or/and as «self» damage signals that initiate and reinforce inflammation and affect the direction of the adaptive immune response. To sum up, the findings collected in the review allow us to classify the coagulation factors, such as Damage-Associated Molecular Patterns that break down the conventional concepts of the coagulation system.

## 1. Introduction

Bloodstream invasion by pathogenic microorganisms poses a lethal threat to hosts; therefore, to limit pathogen dissemination, an infection is suppressed by all available means, including immunity and thrombotic mechanism activation. When protective structures are formed and the pathogen is destroyed, remodeling/repair processes are initiated [1]. Leukocytes are involved in all stages of pathogen elimination, thrombus formation, and tissue regeneration. Therefore, it is not surprising that there are numerous crosslinks between coagulation and the immune system [2]. The blood and hemolymphs are both unique compartments wherein the components of the two easily activated defense systems are located at a high concentration and in close proximity.

In lower organisms, immune reactions and hemostasis are carried out by the same cells, known as hemocytes. Moreover, it is believed that it was from the hemocytes in lower organisms that both the immune system and the blood clotting system evolved [3]. Hemocytes can identify foreign organisms with a number of pattern recognition receptors (PRRs) and scavenger receptors [4]. Upon activation, hemocytes rapidly degranulate and release antimicrobial peptides, such as the anti-lipopolysaccharide (LPS) factor, tachyplesin, and Big defensins [5,6,7]. These molecules opsonize, isolate, and kill invading pathogens. Apart from their excellent phagocytic ability [8], hemocytes produce blood clotting factors C and G, which interact with hemolymph proteins in a calcium-dependent manner, triggering coagulation [3]. Soluble protein and hemocyte receptor cross-binding results in the cellular aggregation that prevents bleeding and restrains pathogen dissemination [9].

The mutual activation of inflammation and thrombosis does not pose a particular problem for lower organisms because of their open circulatory system, so the main danger they face in the case of an injury is body fluid loss [10]. By contrast, due to the development of the closed circulatory system in higher organisms, thrombosis becomes a challenge, which is sorted out in the following way—the bloodstream turns into a sort of immune privileged organ. The conditions that provoke the activation of the coagulation or inflammation are highly regulated with endothelial cells, producing a range of anticoagulation/anti-inflammatory factors, so the relationship between hemostasis and inflammation is not that obvious [11]. However, in a number of pathologies, such as sepsis [12], ischemia/reperfusion injury [13], and alterations in graft function [14], endothelial homeostatic mechanisms are disrupted, and a mutual reinforcement feedback loop forms between the inflammation and coagulation sites [15]. Moreover, coagulation-mediated inflammation is difficult to restrain in tissues as the concentration of anticoagulation/anti-inflammatory factors is not as high as in the bloodstream (tumor [16], neuroinflammation [17], arthritis [18], and lungs [19]).

The aim of this review was to consider the coagulation system mechanisms as an integral part of the immune system. We analyzed articles in PubMed, Scopus, and Embase. The data were searched for using the keywords “coagulation”, “immunity”, “Factor XII”, “Thrombin”, “Fibrinogen”, “inflammation”, “PAMPs”, “DAMPs”, “PRRs”, “uPAR”, and “PAR”. This review includes research data published over the past 30 years.

## 2. Coagulation Cascade in Mammals

**Figure 1:**
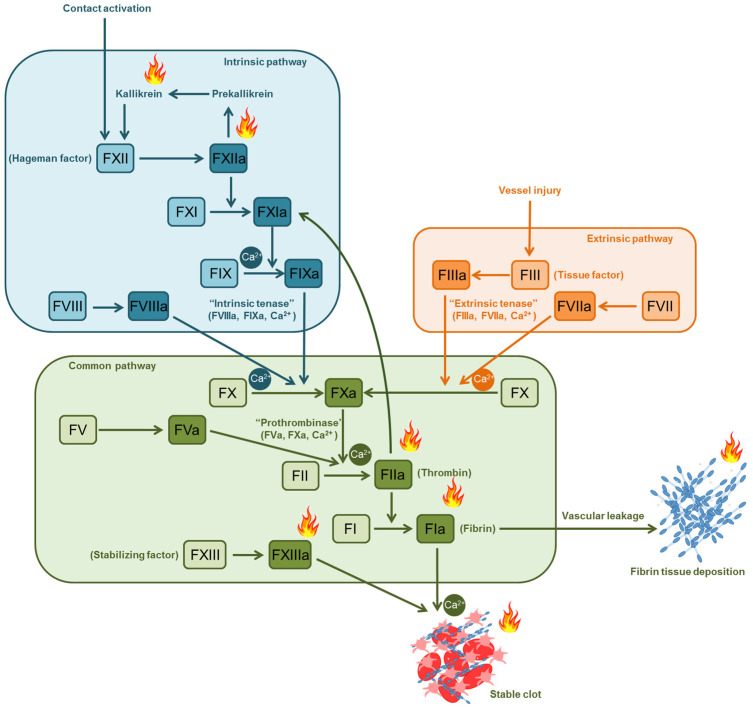
The mammalian blood coagulation network. The "fire" icon indicates the components of the coagulation system involved in the development of inflammation. See details in the text.

Traditionally, the mammalian coagulation system is divided into extrinsic and intrinsic pathways (Figure 1). Each of these cascades leads to the formation of the extrinsic (tissue factor (TF): Factor (F) VIIa, and the intrinsic (FIXa:FVIIIa) tenase complex, both catalyzing the formation of the prothrombinase complex (FXa:FVa) and, further, thrombin. The classical cascade coagulation model describes separately the activation of the intrinsic and extrinsic pathways, which then turn into a common pathway (FX, FV, FII) [20]. The coagulation cascade is described in detail in the reviews [21,22].

### 2.1. The Extrinsic Coagulation Pathway Is Triggered as a Result of Inflammation

The extrinsic blood clotting pathway is initiated by exposure of the TF, which is expressed in the subendothelial tissue and activated leukocytes, mainly monocytes. The TF connects coagulation and inflammation in the protective response to injury (Damage-Associated Molecular Patterns (DAMPs)) or pathogenic microorganism invasion (Pathogen-Associated Molecular Patterns (PAMPs)). The cross-interactions between the blood clotting system and inflammation are almost always mediated by this key receptor.

The TF is a type I membrane glycoprotein that belongs to the interferon receptor superfamily, known as type II cytokine receptors [23] (Figure 1). The TF is found in subendothelial cells in abundance, and have particularly high levels in the brain, skin, lungs, intestines, and placenta. The vascular endothelium minimizes contacts between tissue and plasma procoagulant proteins. Under physiological conditions, leukocytes do not express the TF, but the latter increases rapidly in response to chemical or physical damage, inflammatory cytokines, PAMPs, and DAMPs [24,25]. Thus, in the case of vascular damage and/or the induction of inflammation (events that are difficult to separate), the TF becomes available to proteins of the plasma coagulation system. The TF molds a complex with a small amount of circulating Factor VIIa (FVIIa) [26], the extrinsic tenase complex TF:FVIIa, which activates FX. FXa then binds prothrombin (FII) to form thrombin (FIIa), which, however, can be effectively terminated by TF pathway inhibitors [20]. The amount of thrombin produced may be sufficient to activate platelets and form fibrin clots only following severe damage with prolonged perivascular TF exposure [27].

### 2.2. The Intrinsic Coagulation Pathway Is Triggered by the Direct Recognition of Pathogenic Patterns and Damaged «self»

**Figure 2:**
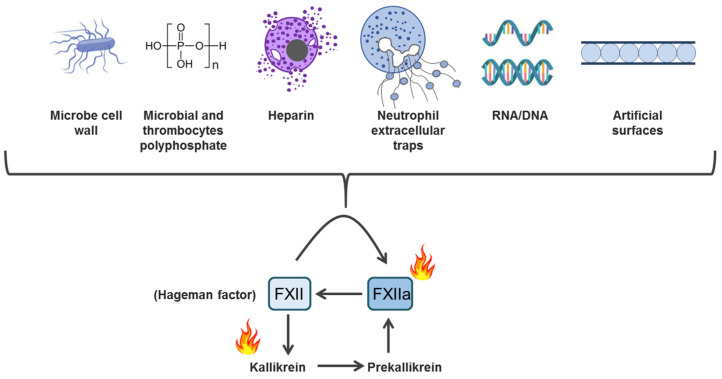
FXII as a soluble receptor for recognition «foreign» and damaged «self». The "fire" icon indicates the components of the coagulation system involved in the development of inflammation. See details in the text.

The intrinsic coagulation pathway is a proteolytic cascade initiated by the activation of the zymogen Factor XII (FXII), Hageman factoris a glycoprotein with a molecular weight of about 90 kDa. FXII has a unique ability to be autoactivated on artificial or biological surfaces that bear undefined signs of foreignness (Figure 2).

FXIIa proteolytically cleaves plasma precallikrein (PK), and then the resulting plasma kallikrein (pKa) releases the nanopeptide bradykinin (BK) from high-molecular-weight kininogen (HK). pKa and FXIIa mutually enhance each other’s proteolytic activation [28,29]. Autoactivation, reciprocal activation, and amplification are the essence of the contact system. When zymogen is incubated along with a negatively charged surface, FXII autoactivation proceeds slowly, but it is significantly accelerated when FXII is incubated with plasma, high-molecular-weight kininogen (HK), or precallikrein (PK) [30]. FXII activation by plasma kallikrein creates conditions allowing FXIIa to form more PK, thus enhancing the system’s productivity (Figure 2), while HK stabilizes PK [28,29]. Tissue injury, resulting in DAMP accumulation, also contributes to the FXII and FXI-mediated activation of the contact pathway, providing a procoagulant and prothrombotic background [31]. Vascular damage and the exposure of extracellular matrix proteins, such as laminin and collagen, also activate FXII [32,33].

Conformational changes in FXII bring about its autocatalytic cleavage and the formation of the active serine protease. Next, the FXIIa-controlled sequential activation of FXI and FIX occurs, which, interacting with its cofactor (FVIII), forms a tenase complex (FIXa:FVIIIa) to activate FX [27].

According to modern concepts, the intrinsic pathway serves to enhance the production of thrombin, generated through the extrinsic TF pathway [34]. Thrombin cleaves FXI, then, following FXIa and FIXa, contribute to increased thrombin production along the “truncated” intrinsic pathway (Figure 1). Provided that there is an excessive amount of the TF, this mechanism is probably unnecessary, but with a limited amount of TF, FX activation, due to the intrinsic pathway, causes rapid thrombin formation sufficient for the formation of a hemostatic plug [35,36].

## 3. The Role of Hemostasis Activation in Inflammatory Diseases

Numerous clinical observations show that, apart from its physiological role, the activation of the hemostasis system underlies the pathogenesis of diseases of various origins. The pathogenic role of hemostasis factors has been proven in pathological thrombosis, neuroinflammation, rheumatoid arthritis (RA), allergic diseases, tumor growth, bacterial infections, and viral infections (Table 1).

### 3.1. Infection Diseases

In various viral infections, the hemostasis system is triggered both directly, due to the cytopathic action of the pathogen on endotheliocytes, and indirectly, due to the release of PAMPs and DAMPs. In both cases, the activation of the contact and extrinsic coagulation pathways occurs. Among the significant triggers associated with contact pathway activation during a viral infection, there is a release of molecular structures caused by host cell death. Extracellular RNA, released by necrotized cells, binds to FXII and FXI, contributing to the formation of a procoagulant and prothrombotic environment [31]. The activation of FXII, supporting blood clot formation, is also caused a local or systemic vascular endothelial damage, which provides FXII access to extracellular matrix proteins, such as laminin and collagen [31]. The activation of neutrophils with neutrophil extracellular trap (NET) release, induced by various stimuli, is another mechanism for FXII activation and contact pathway triggering. At the same time, recent studies have shown that, in viral pneumonia, a wide range of cells, including bronchial epithelial cells, neutrophils, monocytes, macrophages, endothelial cells, and adventitious fibroblasts, begin to express the TF [37]. The TF contributes to extrinsic coagulation pathway initiation and NET formation in acute viral infections, such as Coronavirus Disease-2019 (COVID-19) [37,78]. A number of studies have shown the release of NETs after neutrophil activation by viruses, including Human Immunodeficiency Virus (HIV), influenza, Dengue Virus (DENV), Chikungunya Virus (CHIKV), Respiratory Syncytial Virus (RSV), Hantavirus (HTNV), and Severe Acute Respiratory Syndrome Coronavirus (SARS-CoV)-2 [31].

An in vitro study proved that the infection of Human Umbilical Vein Endothelial Cells (HUVECs) with the pathogenic strain of HTNV in the presence of FXII and PK leads to the increased cleavage of HK and BK [79]. The addition of components of the kallikrein–kinin system to infected cell cultures also results in an increase in endothelial permeability, suppressed by FXIIa inhibitors, as well as B2R antagonists. Clinical studies using B2R antagonists have shown the partial elimination of respiratory symptoms [80]. Thus, van de Veerdonk et al. showed that eight out of nine patients (89%) with COVID-19 requiring supplemental oxygen experienced a decrease in oxygen delivery of 3 L/min or more after 24 h following the administration of icatibant (a selective bradykinin receptor type 2 antagonist) [80]. Moreover, in 1975, Edelman and his colleagues demonstrated a significant decrease in the levels of zymogens FXII and PK in the blood plasma of children with Hemorrhagic Dengue Fever (DHF) against patients with unrelated fevers [81].

A relation was established between the intrinsic pathway of the coagulation system and neutrophil activation in COVID-19 patients [82]. It has been shown that NETs are colocalized with FXIIa in sites of microthrombi [83]. The analysis of blood plasma from patients with COVID-19 demonstrated an increase in cytokines that stimulated the formation of NETs IL-6 (53.49 ± 10.91 vs. 0.3626 ± 0.2055 pg/mL in healthy donors) and IL-8 (17.52 ± 12.26 vs. 4.236 ± 1.280 pg/mL in healthy donors) [83]. FXIIa concentration in bronchoalveolr lavage fluid in Acute Respiratory Distress Syndrome (ARDS) patients was significantly higher in non-survivors compared with survivors [84]. FXIIa increased the expression of inflammatory cytokines interleukin (IL)-8, IL-1β, IL-6, CXCL5, Leukemia Inhibitory Factor (LIF), and Tumor Necrosis Factor (TNF) α, independently on pKa [85].

Elevated concentrations of tissue pKa, BK, and myeloperoxidase complexes with DNA (MPO–DNA) were also recorded in the blood of patients with COVID-19 [86]. Martens et al. showed increased levels of the kinin peptide bradykinin-(1–5) (89.6 pM vs. 0.0 pM in the control group), MPO-DNA complexes (699.0 ng/mL vs. 70.5 ng/mL in the control group), and increased tissue kallikrein activity (18.2 pM vs. 3.8 pM in the control group) in the bronchoalveolar lavage fluid of patients with severe COVID-19 [86].

Kallikrein–kinin system activation can be caused by the expression or accumulation of viral proteins in host cells. In an in vitro study, it was demonstrated that recombinant structural proteins such as SARS-CoV-2 (proteins S, M, and N), bound to PK and FXII and induced BK formation during incubation with plasma from healthy donors [87].

Enzymes or negatively charged molecules, released by mast cells, are considered as potential candidates causing the activation of the kallikrein–kinin system during SARS-CoV-2 infection [88]. Recently, Englert at al. demonstrated an increased frequency of FXIIa in plasma and lungs tissue and its colocalization with NETs in patients with COVID-19. The authors suggested that the increased infiltration and activation of neutrophils with impaired NET clearance contributed to FXIIa formation in this pathology [89]. In another study, elevated levels of kinin peptides and MPO–DNA complexes (considered as NET markers) in the blood of patients with COVID-19 were shown [86]. In hospitalized patients with COVID-19, a significant increase in the levels of intact and cleaved kininogen in blood plasma, as well as kallikrein:C1 esterase inhibitor (C1INH) and FXIIa:C1INH complexes were found, as evidence of the kallikrein–kinin system’s activation [90]. An increased BK1-8/BK ratio was associated with the elevated production of inflammatory cytokines (IL-6, TNF, and IL-1β), blood clotting markers (D-dimer, fibrinogen, and TF), and lymphopenia, and correlated with disease severity in patients with the rhinovirus and influenza virus [91]. It has been found that thrombin formation, especially along the extrinsic pathway, is crucial for the development of COVID-19 infection. Thrombin-mediated vascular damage is an important indicator of disease severity, thrombosis, and mortality [92].

As mentioned previously, in COVID-19 patients, the extrinsic coagulation pathway is also triggered in a TF-dependent manner. Skendros et al. demonstrated high levels of circulating TF in the plasma of patients with SARS-CoV-2 [78]. Moreover, peripheral blood neutrophils in patients with COVID-19, expressing the TF, induced the thrombotic activity of Human Aortic Endothelial Cell Culture (HAEC). The authors also showed NETosis to increase thrombogenicity [78].

Other works have demonstrated the increased expression of the fibrinogen receptor Macrophage-1 antigen (Mac-1) in monocytes of patients with severe COVID-19 [93]. Platelets from the blood of those patients were able to bind to monocytes, upregulate TNFα, and cause IL-1β secretion and inflammation in a TF-mediated way [93]. Similar results, obtained by Goonewardena et al., show a relation between the monocyte TF level and the severity of the disease. The authors also demonstrated the activation of monocytes obtained from conditionally healthy donor blood under the influence of serum from patients with severe COVID-19 [94]. It was found that TF expression in the lungs of patients with COVID-19 ARDS was significantly higher compared to both healthy donors and patients with another ARDS etiology (bacteria, influenza, and aspiration). An immunohistochemical study of the lung tissues of patients with COVID-19 clearly showed fibrin thrombi and thrombi expressing platelet factor (PF) 4 to co-localize with the TF [95]. The peripheral blood of the patients with severe COVID-19 contained 231 (39;761) fM extracellular vesicle (EV) TF, while in patients with moderate COVID-19—25 (12;59) fM. In turn, patients with thromboembolic complications had an EV-TF concentration of 629 (79;1104) fM, while in patients without these complications—34 (14;105) fM EV-TF [95]. There was also an increase in the EV-TF level and thromboembolic events in patients with severe COVID-19 compared to moderate-COVID-19 ones [96]. Thus, recent studies have shown the involvement of both contact and extrinsic hemostasis pathways in the initiation of immunothrombosis in an acute coronavirus-infection patients.

There is also a relationship between hemostasis and inflammation reactions in bacterial infections. Severe bacterial sepsis often leads to a systemic clotting disorder and a proinflammatory condition, which can manifest as disseminated intravascular coagulation (DIC) syndrome, septic shock, and multiple organ failure [38]. In the studies showing FXI’s role in the development of peritoneal sepsis in mice, it was shown that early anticoagulant therapy with the drug 14E11 (an antibody that blocks FXI activation under FXIIa) suppressed the formation of the thrombin–antithrombin complex, IL-6 and TNFα levels, and reduced platelet activation [38]. The administration of the 14E11 antibody for 12 h after sepsis induction in mice significantly improved their survival rate when compared with the control group, and the saturating dose of the drug did not affect blood clotting. These data indicate that severe polymicrobial abdominal infection is accompanied by prothrombotic FXI activation [38]. Similar results were obtained in a study of 14E11 being administered to primates [97]. Premedication of baboons with the 3G3 antibody, a humanized version of the mouse antibody 14E11, significantly reduced the activation of coagulation, which affected the clotting time and plasma complexes of coagulation proteases (FXIIa, FXIa, FIXa, FXa, FVIIa, and thrombin) with serpins—C1INH and antithrombin. After the infusion of heat-inactivated *S. aureus,* 3G3 antibody treatment decreased fibrinogen and platelet consumption, fibrin tissue deposition, tissue neutrophil activation and accumulation, cytokine production, kininogen cleavage, complement activation, and cell death [97]. In bacterial sepsis, there is an increased expression of the TF on peripheral blood monocytes as evidence of extrinsic hemostasis pathway activation and the development of immunothrombosis [12].

A predisposition to hypercoagulation was also recorded in the Human Immunodeficiency Virus (HIV) infection [44]. An increase in the Plasminogen Activator Inhibitor-1 (PAI-1) level in the peripheral blood of patients with HIV infection was shown [44]. Moreover, the level of PAI-1 positively correlated with a viral load, which might reflect the relation of a retroviral infection with coagulation activation. Also, protein S, a coagulation inhibitor, increased by the sixth month of antiretroviral therapy (45.53 ± 14.64% before vs. 54.9 ± 16.05% after therapy), while protein C and Antithrombin III only tended to increase by 75.40 ± 22.76% before vs. 85.37 ± 27.8% after therapy, and 104 ± 17.54% before vs. 110.53 ± 14.92% after therapy, respectively [44]. The study by Bhoelan et al. showed a decrease in immune activation, normalization of the CD4/CD8 ratio, and a reduced procoagulant state in HIV patients during the first year of successful antiretroviral therapy [42].

### 3.2. Cardiovascular Diseases

Recent studies have demonstrated a close association of FXII activation with pathological thrombosis. Endogenous Polymeric Phosphates (polyPs) on activated platelets or the intravenous administration of exogenous polyPs led to FXIIa accumulation and fatal pulmonary embolism development in wild-type mice. In contrast, a genetic deficiency of FXIII or FXIIa prevented pathological thrombosis [98,99]. The recombinant contact phase inhibitor from *Ixodes ricinus* mites, the Kunitz-type protein (Ir-CPI), interacts with human FXIIa, FXIa, and pKa, and results in the extension of thromboplastin time in vitro [98]. Intravenous Ir-CPI administration saved rats and mice from venous thrombosis in a dose-dependent manner [98]. A decrease in neutrophil migration to the inflammatory site was associated with a low number of NETs and improved wound healing in a model of sterile inflammation in mice with an FXII deficiency (F12−/−) [100].

It is worth noting that an increased level of FXIIa is associated with hypercholesterolemia and hypertriglyceridemia [46], metabolic disorders characteristic of insulin resistance. It was shown that the administration of a direct thrombin inhibitor, argatroban, protected animals against the development of glucose intolerance, cardiac fibrosis, and inflammation by suppressing nuclear factor κB (NF-κB) and caspase-3 activation in a rat model of diabetes mellitus type 2 [45]. These studies also confirm the involvement of hemostasis in the inflammatory origin of insulin resistance.

Lately, evidence of the possible engagement of the coagulation cascade in the pathogenesis of chronic autoimmune urticaria is presented [47,48,49,50]. Cugno et al. demonstrated an increase in plasma prothrombin in patients with urticaria (3.06 nmol/L vs. 0.80 nmol/L in the control group). Moreover, the prothrombin level was directly related to the severity of the disease: (r = 0.37; *p* < 0.05) 65 [47,48,49,50].

Multiplex analysis based on the immuno-polymerase chain reaction makes it possible, purposefully and efficiently, to determine the expression of a relatively large number of plasma proteins. Using this technology, the relationship between 444 blood plasma protein expression and FXIa in patients has been studied for 12 months since acute venous thromboembolism arose. Several FXIa-related markers that play a role in T-cell activation and chemotaxis (CD28, CCL16, CCL23, CCL5, and Thymic Stromal Lymphopoietin (TSLP)), inflammatory mediators (IL-1α, Pentraxin-related protein (PTX3), IL-18, IL-17C, and IL-27), and protective proteins (Regenerating family member 3 Alpha (REG3A), Defensin Alpha 1 (DEFA1), Chitinase 1 (CHIT1), and Cathepsin Z (CTSZ)) were found. Still, the fine mechanisms of FXIa’s influence on immune processes require further study [101].

Thrombin is believed to contribute not only to the initiation, but also to the progression of atherosclerotic lesions. This is confirmed by the studies on a murine model, as the removal of the natural thrombin inhibitor aggravated the atherogenic condition. On the contrary, a decrease in thrombin activity weakened the progression of plaques and contributed to their stability in advanced atherosclerotic lesions [102]. The administration of direct thrombin inhibitors, such as hirudin, argatroban, and dabigatran, reduced circulating levels of inflammatory markers and leukocyte recruitment, thereby slowing the progression of the disease in animal models of atherosclerosis and arterial restenosis [103].

An increased fibrinogen blood level is considered to be a marker of a high risk of cardiovascular diseases, such as hypertension and atherosclerosis [104]. The elevation of fibrinogen and other hemostasis factors (von Willebrand, Tissue-Type Plasminogen Activator (tPA), PAI-1, FVII, and FVIII) are associated with the development of atherosclerosis and cardiovascular diseases [105]. The epidemiological data confirm the causative relation between fibrinogen production and primary cardiovascular disease development, which makes it one of the most reliable risk factors, along with smoking, hypertension, diabetes mellitus, and hyperlipidemia [106].

High levels of fibrinogen-breakdown products, such as D-dimers, are widely used in clinical practice as indicators of inflammation, markers of increased blood clotting, and predictors of thrombotic complication development [104].

### 3.3. Neuroinflammation

Blood coagulation cascade components are the key mediators of neuroinflammation development. Gobel et al., in studies on the role of FXII [53] in an experimental model of multiple sclerosis, showed that FXII−/− mice are less prone to developing central nervous system inflammation. The pharmacological blockade of FXIIa protected against Experimental Autoimmune Encephalomyelitis (EAE) (a model of multiple sclerosis), regardless of FXIa and kallikrein–kinin formation. FXIIa stimulated Th17 lymphocyte polarization in Plasminogen Activator, Urokinase Receptor (uPAR)-dependent manner [53]. The deficiency and pharmacological blockade of FXII make animals less susceptible to EAE and are accompanied by a decrease in the number of T cells producing IL-17A. The FXII-induced activation of adaptive immunity is mediated by uPAR on dendritic cells. High levels of FXIIa are recorded in the plasma of patients with multiple sclerosis during relapse. The inhibition of FXIIa may provide a strategy to combat multiple sclerosis, as well as other disorders associated with autoimmune nerve-fiber damage [53].

Thrombin is involved in the pathogenesis of inflammatory brain diseases, such as multiple sclerosis [51] and, possibly, Alzheimer’s disease [107,108]. It is assumed that uncontrolled inflammation, triggered by thrombin through Protease-Activated Receptor (PAR) activation, contributes to the damage of neuronal tissue, disrupts electrophysiology, blocks of nerve conduction, or seizures [51,109]. It has been shown that thrombin [110,111] induces astrogliosis [112,113], demyelination [114], and a number of other neurotoxic processes [115,116]. The use of specific thrombin inhibitors significantly reduces inflammation and promotes the functional restoration of nerve fibers after damage to the central nervous system [117,118,119]. The positive role of thrombin inhibitors in neuroinflammation is, at least, partially explained by the suppression of IL-1α production [120]. The suppression of thrombin also significantly reduces macrophage Migration Inhibitory Factor (MIF) production and promotes functional recovery in experimental animals [121]. The administration of hirudin in EAE significantly decreased the disease severity and suppressed T helper cell (Th) 1 and Th17-derived cytokine production [122].

Under physiological conditions, due to the complex structure of the vascular walls in the central nervous system, forming the Blood–Brain Barrier (BBB), plasma fibrinogen does not enter nervous tissue. However, some pathological conditions associated with either acute hemorrhage (brain or spinal cord injury and hemorrhagic stroke) or chronic BBB disorder (multiple sclerosis, Alzheimer’s disease, glioblastoma of the brain, HIV encephalitis, and bacterial meningitis) lead to fibrin deposition in the central nervous system [39,40,41,43]. BBB disruption, fibrin deposition, and microglia activation are the earliest histopathological signs that can be detected even in normal white matter without signs of demyelination [123,124]. Increased inflammatory activity, as well as pronounced microglia activation, correlates with fibrin deposition in active demyelinating lesions in multiple sclerosis [52,54]. The importance of the adhesion molecule CD11b/CD18 and fibrinogen interaction for its proinflammatory effect on microglia was demonstrated [125,126,127].

Traumatic brain injury (TBI) causes a significant BBB breakdown, resulting in the extravasation of blood proteins into brain tissue. Cerebral fibrinogen deposits bring about innate immune cell activation in both human and murine TBIs. The abrogation of the fibrin–CD11b interaction reduces tissue inflammation after a TBI [17].

### 3.4. Autoimmune Diseases

Animal models have shown that pain, edema, and fibrin deposition are the most noticeable disease manifestations during the first weeks after rheumatoid arthritis induction [128]. It was found that fibrinogen induced the NF-κB-dependent secretion of IL-8, Growth-Regulated Oncogene Alpha (GROα) and the expression of intercellular adhesion molecule (ICAM)-1 in synovial fibroblasts [129]. Fibrin breakdown products, such as D-dimer, are common biomarkers found in blood plasma as well as in synovial fluid in patients with rheumatoid arthritis [56,61,63]. Ingegnoli et al. showed an increase in prothrombin concentration in the peripheral blood of patients with RA of up to 339 pmol/L (250–894 pmol/L) vs. 159 pmol/L (118–195 pmol/L) in the control group. In addition, such patients showed an increase in D-dimer levels in the peripheral blood of up to 1706 ng/mL (937–3518 ng/mL) vs. 254 ng/mL (174–288 ng/mL) in the control group. At the same time, the 14-week treatment of patients with RA with a TNF-alpha inhibitor (infliximab) showed a decrease in prothrombin (189 pmol/L (69–224 pmol/L) and D-dimer (752 ng/mL (507–1052 ng/mL)) [56]. So et al. showed an increased level of procoagulant factors in the synovial fluid of patients with inflammatory arthropathy. For example, the concentration of the tissue factor in the synovial fluid of patients with rheumatoid arthritis was 12.6 ± 1.3 pmol/L^−1^, spondyloarthritis was 12.4 ± 3.1 pmol/L^−1^, and crystalline arthritis was 13.8 ± 2.9 pmol/L^−1^, while, in the control group of patients with osteoarthritis, the concentration of the tissue factor in synovial fluid was 5.82 ± 0.89 pmol/L^−1^ [61]. In the model of antigen-induced arthritis, synovial cavity fibrin deposition correlates with the aggregation and activation of intima cells [59]. Fibrin accumulation on cartilage correlated with the severity of tissue damage in explants of patients with RA and in adjuvant-induced arthritis in mice [18].

Thrombin can also cause adverse reactions associated with the pathophysiology of rheumatoid arthritis [58]. Thrombin inhibition suppresses synovial inflammation and improves the condition, even in a long-running process [57,62]. Thrombin leads to increased PAR-1 expression in an inflamed joint and, at the same time, serves as an inducer of PAR-1-mediated cell hyperproliferation and inflammation [55,60].

Studies of patients with antiphospholipid syndrome have also demonstrated that increased TF expressions on endotheliocytes and neutrophils bring about thromboembolic events [64]. Moreover, endothelial cell proliferation, often observed in this pathology, leads to occlusive vasculopathy, contributing to organ failure [64].

### 3.5. Allergic Deseases

Thrombin has been found to play an important role in the development of type 2 inflammation. Particularly, thrombin is involved in the generation of IL-33. Low-molecular-weight h eparin (LMWH), an indirect thrombin inhibitor, suppresses allergic immune reactions induced by papain and fungi in mice by inhibiting IL-33 generation [130].The activation of the blood coagulation system and increased thrombin production take part in remodeling upper respiratory tract tissues in allergic processes [66]. Thrombin can stimulate the production of IL-6, IL-8, prostaglandin (PG) E2, CCL2, Platelet Growth Factor (PDGF), and Mucin 5 Subtype AC in the epithelial cells and, thus, affect epithelium permeability and eosinophils migration into the respiratory tract [131,132,133,134,135,136,137]. Blocking or reducing the thrombin-mediated activation of PAR1 also suppresses lung inflammation and fibrosis in mice [138]. In the research by Shimizu et al. [139], the presence of thrombin and the thrombin–antithrombin complex in the nasal secretions of patients with allergic rhinitis, Chronic Rhinosinusitis with Nasal Polyps (CRSwNPs), and asthma were recorded. A significant local activation of the blood clotting system and thrombin-mediated tissue remodeling in the upper respiratory tract of patients with CRSwNPs were also demonstrated [139]. Thrombin stimulated mucin secretion in human primary bronchus epithelial cells. This effect was imitated by specific PAR-1 stimulation [137]. In accordance with these in vitro observations, intranasally administered thrombin induced goblet cell hypertrophy in the rat nasal epithelium [137]. Thrombin-mediated PAR-1 stimulation caused the proliferation of smooth muscle cells in vitro, promoting the production of PDGF, extracellular matrix proteins [136], and fibrosis development. Bronchoalveolar lavage fluid obtained from patients with atopic bronchial asthma induced fibroblast proliferation in vitro. This effect was suppressed by hirudin [140]. In addition, it has been shown that thrombin can increase the tone of human bronchi in vitro [141].

FXII-mediated bradykinin formation was observed in the plasma of patients with anaphylaxis, as well as in animal experimental models of anaphylaxis. It has also been shown that the severity of anaphylaxis correlates with the intensity of activation of the contact coagulation system [142]

### 3.6. Oncological Diseases

Blood clotting factors in general and thrombin in particular play an important role in cancer immunobiology [69,70,71,72,73,74]. A study of patients with malignant neoplasms of the mammary gland, lungs, gastrointestinal tract, pancreas, kidneys, prostate, and brain, as well as lymphoma, multiple myeloma, and other types of tumors showed an average peak of thrombin concentration of up to 500 nM [68].

Hypercoagulation in tumor growth has serious therapeutic and important diagnostic consequences. At a certain point in tumor progression, procoagulants, produced by tumor cells, enter the bloodstream, followed by the development of thrombosis with serious, often life-threatening consequences. However, thrombosis can also be a warning sign of an undiagnosed “hidden” malignant neoplasm (so-called Trousseau syndrome) [75].

Apart from tumor cell behavior regulation directly through PARs, or indirectly by fibrin generation, thrombin activates effector cells in the immune system and modulates inflammatory processes [67]. The thrombin-mediated release of IL-6, TNFα, and monocyte chemoattractant protein (MCP)-1 results in the accumulation of immune cells in tumor tissue [67]. Platelets activated by thrombin release Transforming Growth Factor-β (TGF-β), which inhibits the natural killer cell activity and promotes the differentiation of myeloid suppressor cells, M2-like macrophages, and regulatory T cells (Treg), helping the tumor to evade host immune control. Removing PAR-1 from the tumor microenvironment, but not from the tumor itself, significantly reduces its growth and metastasis [67].

### 3.7. Other Diseases

FXII upregulation in kidney tubular cells correlates with the organ dysfunction in diabetic kidney disease patients. The uPAR-mediated FXII interaction with tubular cells induces oxidative stress, DNA damage, and senescence. uPAR or β1-integrin blocking ameliorates FXII-induced tubular cell injury [76].

Pain, hypercoagulation, and chronic inflammation are the most common complication of sickle cell disease (SCD). Patients with SCD exhibit an increase in circulating biomarkers of FXII and contact pathway activation, compared with healthy controls. FXII contributed to enhanced TNFα-induced inflammation and thrombin generation in sickle cell mice. FXII inhibition significantly reduced venous thrombosis, microvascular stasis, and attenuated brain damage after induced ischemia/reperfusion in sickle cell mice [77].

## 4. Current Concept of Triggering Inflammation and an Immune Response

Presently, the most generally accepted concept, explaining the initiation mechanisms of protective immune reactions, is the one by Polly Matzinger. It states that immune cells respond to invading microorganisms primarily because of tissue damage and cellular stress. Specific molecules, released from dying cells and activating innate immunity, have been designated as damage or DAMPs. The research into this group of molecules resulted in a deeper understanding of the processes underlying non-infectious inflammatory diseases [143]. DAMPs are a rather heterogeneous group of molecules combined, however, by two common features; they have an endogenous origin and trigger innate immune responses. According to the latest classification, DAMPs are divided into constitutive (c)DAMPs and inducible (i)DAMPs [144]. cDAMPs are intracellular molecules that remain unchanged when cells are damaged. iDAMPs are formed only in dying cells and released after their destruction [145]. Recently, DAMPs, the formation of which is characteristic of a certain lifestyle, Lifestyle-Associated Molecular Patterns (LAMPs), have been isolated into a separate group, including cholesterol, urate, and Oxidized Low-Density Lipoproteins (OxLDLs) [144]. A distinctive feature of LAMPs is that they are not excreted from the body, so their accumulation results in chronic inflammation. Despite the fact that DAMPs differ greatly in their origin and functions, they are all recognized by a large group of PRRs as well as non-PRRs, which include Receptors for Advanced Glycation End product (RAGEs) 7, Triggering Receptors Expressed on Myeloid cells (TREMs), several G-Protein-Coupled Receptors (GPCRs), and ion channels [143]. DAMPs’ recognition by their cognate receptors drives an inflammatory response, developing in the same way: the release of cytokines, recruitment of leukocytes, and activation of adaptive immune reactions [144]. DAMPs can cause specific immune responses that are not necessarily destructive, but regenerating [146]. Moreover, a reasonable production of DAMPs is necessary to achieve the complete restoration of homeostasis and regeneration after tissue damage [147]. Components of the coagulation cascade exhibit all the features characteristic of PRRs as well as (i)DAMPs.

## 5. Mechanisms of Immune System Activation under the Influence of Coagulation Factors

### 5.1. Innate Immunity

**Figure 3:**
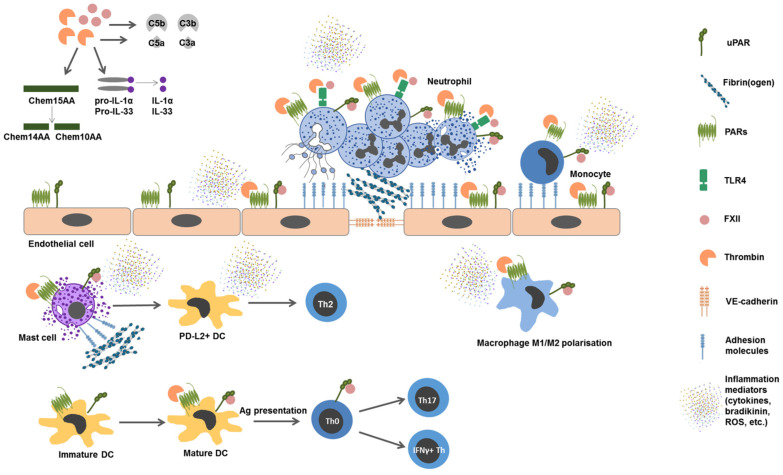
Activation of innate and adaptive immunity by coagulation factors.

Of all the coagulation factors, the inflammatory effects of FXII, thrombin (FII), and fibrin(ogen) have been the most well-documented (Figure 3, Table 2).

The activation of the coagulation system leads to the production of chemoattractants (anaphylotoxins and active fragments of chemerin), which attract leukocytes to the injured site. The components of the coagulation cascade cause a receptor-mediated (uPAR, PARs, TLR-4, and adhesion molecules) activation of the vascular endothelium and immune cells and enhance the adhesion and transmigration of leukocytes into tissues. The enzymatic activity of FIIa leads to IL-1β and IL-33 alarmin generation that increases inflammation. FXIIa and FIIa activate monocytes and trigger neutrophil degranulation and NET release. Under the influence of coagulation factors, activated endothelial and immune cells produce numerous inflammatory mediators (ROS, bradykinin, prostaglandins, and cytokines). FXIIa and FIIa also stimulate the development of adaptive immunity reactions, contributing to DC maturation and their migration to lymph nodes for antigen presentation. In addition, FXIIa and FIIa can directly and/or indirectly influence the direction of the adaptive immune response due to the polarization of T helper cells and macrophages. See details in Table 2. Abbreviations: Ag, antigen; Chem, chemerin; DC, dendritic cell; FXIIa, activated Factor XII; IL, interleukin; PARs, protease-activated receptors; PD-L2, programmed cell death 1 ligand 2; ROS, reactive oxygen species; Th, T helper cell; TLR, Toll-like receptor; uPAR, urokinase plasminogen Activator receptor; VE-cadherin, vascular endothelial cadherin.

FXII is directly bound to most pathogen surfaces (bacteria, fungi, and viruses). Moreover, RNA, DNA, β-amyloid, denatured proteins, exosomes, polymers of activated platelets, and NETs are among its ligands (Figure 2). The mechanism by which microorganisms can activate FXII involves contact with polyPs, similar to those secreting activated platelets [99,201]. Microbes contain PolyPs ranging in size from hundreds to thousands of phosphate units [202]. PolyPs form calcium ion-rich nanoparticles that trigger FXII autoactivation, regardless of the polymer length on cell surfaces in vivo [203,204]. Consistent with this, PolyPs from *Salmonella* spp. and *Escherichia coli* demonstrate high efficiency in triggering the contact pathway [99,204]. Waack et al. showed that the metalloprotease CpaA, secreted by *Acinetobacter baumanii*, inactivates FXIIa and, thereby, attenuates essential coagulation and inflammation pathways, allowing the pathogen to disseminate [205]. FXII can also interact with foreign inert materials (prosthetics materials, etc.) [206]. This function of PRRs, the recognition of «foreign» or damaged «self», inherent to the intrinsic coagulation pathway, underlies the development of pathological thrombosis, which evolves as a result of inflammation, but not due to blood loss [207,208,209,210,211,212].

A large number of studies prove the ability of coagulation factors to trigger immune responses. Furthermore, the data that reveal the molecular mechanisms of the interaction between coagulation and the immune system are provided (Figure 3, Table 2).

FXIIa stimulates inflammation by interacting with the uPAR, which is expressed on different types of cells, including hematopoietic [100,213,214] and endothelial. Factor XII stimulates extracellular signal-regulated protein kinases (ERKs) 1/2 and Akt (RAC-alpha serine/threonine protein kinase, protein kinase B alpha), serine-threonine protein kinase through uPAR, integrins, and the Epidermal Growth Factor Receptor (EGFR) to initiate angiogenesis [215]. uPAR is a glycosylphosphatidylinositol-linked protein shown to modify intracellular signaling from integrins and GPCRs [216]. uPAR has a low level of expression on resting T cells that increases rapidly after activation through the T-cell receptor or under the influence of phorbol 12-myristate-13-acetate and inflammatory cytokines (TNFα, interferon (IFN)γ, IL-1, and IL-2) [217,218,219,220,221]. Previously, uPAR was considered to perform a thrombolytic function, however, recent studies have demonstrated its significant immunomodulatory role in inflammation and cancer [216].

It was shown that FXIIa induces the production of inflammatory cytokines in alveolar cells [85,188], monocytes [189], macrophages [197], and dendritic cells [53]. FXIIa stimulates the production of TNFα, IL-1β, IL-12, and IL-6 in bone marrow-derived macrophages. Under the influence of FXIIa, bone marrow-derived antigen-presenting cells secrete inflammatory cytokines and stimulate the differentiation of antigen-specific IFNγ-producing CD4+ T cells. The induction of cytokine expression by FXIIa in macrophages occurs independently of the enzymatic activity of the protease. The effect decreases under phospholipase C treatment, proving that the intracellular FXIIa signal is uPAR-dependent [197]. The exposure of peripheral blood monocytes to FXIIa in the presence LPSs results in the increased production of IL-1 [189].

FXII and neutrophil interaction have been the most thoroughly investigated because of the leukocyte involvement in hemostasis reactions. Both proteins, zymogen FXII and FXIIa, have been found to promote neutrophil degranulation [148]. FXII itself contributes to NET formation. The interaction of FXIIa-uPAR increases aMβ2 integrin expression, which, in turn, induces the mobilization of intracellular calcium (Ca^2+^), histone citrullination, and NET release [152]. NETs trigger FXII autoactivation, the accumulation of activated blood clotting factors, and fibrin formation [222]. NETs accelerate coagulation and promote the creation of a denser, more lysis-resistant clot. Neutrophils, or the mediators they release, can cause FXIa-mediated clotting, unlike NETs, which induce clotting with FXIa-independent mechanisms [223].

It has been proven that uPAR is necessary for optimal neutrophil activation after Toll-like receptor (TLR) 2 stimulation. The expression of TNFα and IL-6 induced by cell activation via TLR2 in uPAR−/− neutrophils was lower than in uPAR+/+ neutrophils. Inhibitor kappa B-alpha (IkB)-α degradation and NF-κB activation did not differ in uPAR−/− and uPAR+/+-neutrophils after TLR2 stimulation. However, uPAR was important for optimal TLR2-mediated p38 Mitogen-Activated Protein Kinase (MAPK) activation. According to the data, lung inflammation and systemic inflammation caused by TLR2 stimulation are lower in uPAR−/− mice compared to the wild type [153].

Neutrophils themselves express FXII, which contributes to their activation in an autocrine manner [100]. However, the FXII-uPAR interaction in vivo is a strictly regulated process, which is restricted by the uPAR level. In resting neutrophils, uPAR is stored preformed in secretory vesicles and specific granules. Cell activation leads to increased PLAUR-uPAR gene expression and uPAR plasma membrane translocation [224]. In addition, the binding of FXIIa to uPAR is Zn^2+^-dependent [100], and rises when the concentration of free Zn^2+^ increases from the initial plasma level of ~20 nM up to the micromolar range [100,225]. It is believed that both neutrophils and activated platelets can be Zn^2+^ sources at the site of inflammation [226,227]. Neutrophils express a rich network of Zn^2+^ transporters and, presumably, can enhance their mobilization into the extracellular compartment during inflammation [228]. This partially explains why the FXII-uPAR interaction on the neutrophil membrane and cell activation do not occur continuously.

Thrombin is the central protease in the coagulation cascade and is among the strongest leukocyte drivers (Figure 3, Table 2). The main mechanism of thrombin action is the proteolytic cleavage of the N-terminal extracellular domain of protease-activated receptors (PARs -1, -3, and -4) that express on immune cells [229]. Since the effect of thrombin is similar in strength to that of PAMPs, DAMPs [230], as well as pro-inflammatory cytokines, some authors consider PARs as “non-classical” PRRs [231,232].

Thrombin induces PAR1/Phospholipase C (PLC)-mediated platelet activation: degranulation [233,234], the release of the Platelet-Activating Factor (PAF), IL-8, (MIF-1α, Regulated upon Activation, Normal T-Cell Expressed, and Presumably Secreted (RANTES), MCP-3, CCL17, CCXL1, CXCL5, and serotonin, as well as P-selectin, fibrinogen receptor GPIIb-IIIa, and CD40 ligand upregulation [235,236,237,238,239,240]. The degranulation of thrombin-treated platelets results in TLR4 and TLR9 membrane exposure [241,242,243] and increases the platelets’ sensitivity to PAMP and DAMP signals [244,245,246].

Thrombin can cause rapid, calcium-dependent mast cell degranulation [195] and IL-6 secretion through the activation of the thrombin receptor and FcƐRI signaling pathway [47]. The treatment of mouse mast cells (P815) with different concentrations of thrombin increased PAR1, PAR2, PAR3, and PAR4 expression, as well as the vascular endothelial growth factor (VEGF), TNFα, IL-6, CCL-2, CXCL-1, and CXCL-5 production. These changes were mediated by IkB-a, Stress-Activated Protein Kinases (SAPKs)/Jun amino-terminal kinases (JNKs), p38, and ERK1/2 signaling molecules [195]. The importance of these mechanisms is supported by the evidence that, in inflammatory bowel disease [247,248] and chronic autoimmune urticaria [47], accompanied by mast cells activation, a significant increase in coagulation was recoded [234].

In the early studies, it was shown that α-thrombin was a chemoattractant for neutrophils, comparable by the effects to formyl-Met-Leu-Phe, a bacteria derivative. The protease effect did not depend on its enzymatic activity [154]. The enzyme-independent activity of thrombin has been confirmed in another study. Heat-denatured thrombin was shown to induce the secretion of inflammatory mediators in macrophages to a lesser extent (for TNFα, the Macrophage Colony-Stimulating Factor (M-CSF) and MCP-1), similar to Macrophage Inflammatory Proteins (MIP-2) or even greater (for RANTES and CXCL10) than native α-thrombin. Thrombin treated with hirudin induced the same level of RANTES and interferon gamma-induced protein (IP)-10 as a heat-inactivated one [190]. These studies showed that the inflammatory activity of thrombin could be fulfilled independently on PARs at the expense of other mechanisms that have not yet been revealed.

Fibrinogen, a downstream thrombin target, is an acute phase protein and one of the most powerful inflammatory factors among all the coagulation system proteins [238]. Fibrinogen is glycoprotein synthesized in the liver with a blood plasma concentration in the range of 1.5-3 g/l, which increases significantly during inflammation [162,164,249,250]. Interstitial fibrin deposition contributes to the development of an inflammatory reaction and is a universal sign of tissue damage [251,252] (Figure 3, Table 2).

Fibrinogen is composed of a complex of three of polypeptide chains (Aa, Bβ, and γ). The chains connected by disulfide bridges at the N-terminus form a central globule, while the C-terminus of Bβ and γ chains forms two external D-domains connected to the E-domain with three polypeptide chains. Thrombin cleaves Aα and Bβ chains at the N-terminus releasing fibrinopeptides A (FpA, Aa1–16) and B (Bβ1–15), exposing polymerization sites, which bind to complementary sites in D domains forming protofibrils. As far as the cellular and molecular mechanisms of fibrin(ogen) function have been studied, its role has evolved from a marker of vascular damage to a multifaceted signaling molecule involved in hemostasis [253], fibrosis [254,255], protection against infection [256], and sterile inflammation [238]. The first evidence of the potential inflammatory role of fibrin(ogen) in vivo was discovered in the 1960s and 1970s, when it was demonstrated to enhance leukocyte adhesion and transendothelial migration [161]. In vitro and in vivo studies have shown that fibrin(ogen) directly stimulates cytokines/chemokines production in endothelial and immune cells. In particular, the expression of mRNA and protein IL-8 increases in fibrin-treated endothelial cells [185]. Fibrin(ogen) increases the production of cytokines (TNFα, IL-1β, and IL-6) [162,163], chemokines (MIP-1 and -2, MCP-1) [164], and ROS in peripheral blood mononuclear cells [165]. Thrombin administration stimulates the accumulation of IL-6 and MCP-1 in the peritoneal cavity of mice in a fibrin-dependent manner. The suppression of fibrinogen expression reduces cytokine production in thioglycolate-threated macrophages. The adhesion of macrophages to peritoneal mesothelial cells is reduced in mice with a fibrinogen deficiency, highlighting the key role of this factor in the formation of inflammatory infiltrate [257].

Studies in vitro have shown fibrin(ogen) to enhance the inflammatory functions of leukocytes due to the activation of MAPK, Protein Kinase C (PKC), and NF-κB signaling cascades [258,259,260,261,262,263]. Fibrin(ogen) modulates leukocyte migration induced by various inflammatory stimuli owing to its ability to interact with a variety of partners: vascular endothelial (VE) cadherin, ICAM-1, aIIbβ3, α5β1, aVβ3, aMβ2, and aXβ2 [238]. β2 integrins aMβ2 (Mac-1, CD11b/CD18, and CR3) and aXβ2 (p150,95, CD11c/CD18) remain among the most important and well-studied fibrin(ogen) receptors on leukocytes [261,264,265]. In the model of *S. aureus*-induced peritonitis in mice, the blocking of the 390–396A aMβ2-binding motif of fibrinogen was shown to suppress leukocyte adhesion. Mice with fibrinogen-γ 390–396A knockout demonstrated a significant decrease in bacterial clearance [266].

It should be emphasized that when fibrinogen is in the form of a soluble monomer, its integrin-binding Arginylglycylaspartic acid (RGD) motif remains hidden [267]. The RGD motif becomes available for numerous ligands, only in immobilized fibrinogen or its polymer. This property allows plasma fibrinogen to remain “invisible” for circulating leukocytes, but becomes an easy target when in blood clots and/or at the site of injury [238].

In a number of studies, it was shown that the fibrin(ogen)-induced expression of chemokines was TLR-4-mediated, since the effect was absent in C3H/HeJ mice expressing the mutant form of the receptor [164]. In TLR4-positive HEK293-CD14-MD2 cells, fibrin(ogen) induces inflammatory mediator (IL-6, TNFα, IL-8, MCP-1, MMP1, and MMP9) upregulation. These data allow us to consider fibrin(ogen) as a classic DAMP [268].

Fibrinogen degradation products, generated during fibrinolysis, are also involved in the modulation of inflammation. The main fibrin-derived products are D and E fragments formed from the C-terminus and N-terminus of the molecules, respectively [269,270]. E-fragments can bind VE-cadherin to endothelial cells [186,271]. The sequence, responsible for this interaction, is further cleaved by plasmin resulting in the formation of a 28 amino acid fragment: Bβ15-42. Bβ15-42, competing with E fragments to bind to VE-cadherin, heparin, [272], and carboxypeptidase M [273], suppresses the pro-inflammatory effects of the latter [274]. The low level of this peptide is detected in healthy human blood, but is elevated in patients undergoing therapeutic fibrinolysis [275]. Bβ15–42 inhibits the activation of Rho kinase and prevents the stress-induced loss of endothelial barrier function, as well as leukocyte transmigration [276]. Bβ15-42 as a potential inhibitor of leukocyte extravasation prevents the development of an inflammatory reaction. However, an excessive generation of Bβ15-42 disrupts blood clot resorption, presumably by inhibiting leukocyte accumulation in the early stages of clot formation [269].

In supraphysiological concentrations, the Bβ15-42 peptide has proven to be effective in preventing homotypic VE-cadherin bond dissociation and microvascular dysfunction in the case of myocardial damage in rodents [274,276], pigs [277], as well as humans with severe Ebola virus disease with vascular leakage [278].

Bβ15-42 administration, by reducing inflammation and vascular permeability, exhibits a protective effect on animal models of acute in animal models of acute and chronic ischemia-reperfusion injury of myocard, kidneys and liver injuries [13,279,280]. Moreover, Bβ15-42 was shown to preserve organ dysfunction in animal models of polymicrobial sepsis and acute lung injury [281,282]. These results highlight the important contribution of fibrinolysis products to the regulation of leukocyte function and inflammatory reaction [238].

FXIIIa is a thrombin-activated transglutaminase that catalyzes the formation of covalent ε-N-(γ-glutamyl)-lysine crosslinking between the residues of target proteins [283]. As mentioned above, the main function of FXIIIa is to stabilize fibrin clots, increase their rigidity, and provide resistance to proteolysis [284,285]. In plasma, FXIII is present as a heterotetramer (FXIII-A2B2) consisting of two A subunits (FXIII-A) with catalytic activity and two B subunits (FXIII-B) that inhibit FXIII-A. Upon the activation of coagulation, thrombin cleaves the activating peptide from FXIII-A, then FXIII-B dissociates in a Ca^2+^-dependent manner, and FXIII-A becomes an active enzyme [286].

FXIIIa is necessary for the immobilization of *S. pyogenes* in the fibrin network to prevent bacteria dissemination. The administration of FXIIIa in the site of infection results in a decrease in the bacteria spreading and shows the protective role of this factor in skin streptococcal infection [287].

There is some evidence of the pro-inflammatory role of FXIII. In particular, FXIIIa is used as a histological marker of inflammatory and malignant skin diseases [286], and as a marker of alternatively activated macrophages as well [286]. Immunohistochemical studies revealed FXIII intracellular localization (cFXIII) in various cell types, including monocytes, some populations of macrophages, and dendritic cells [200,288,289]. It has been shown that cFXIII can be activated with an increase in intracellular Ca^2+^ without proteolytic cleavage [290,291] and regulates the functional activity of myeloid cells. Monocytes isolated from patients with FXIIIa deficiency showed impaired FcγR and complement receptor-mediated phagocytic activity [194]. Dendritic cells with a cFXIII deficiency had a chemotactic response to CCL19 and reduced migration to lymph nodes [200]. The binding of FXIIIa to the AT1 receptor on human monocytes increased their adhesion to endothelial cells [292]. Finally, FXIIIa has been shown to support osteoclast differentiation and promote bone resorption [293].

The substrates of FXIIa and FIIa outside the coagulation system present a number of humoral immune factors involved in leukocyte activation, inflammatory infiltrate formation, and pathogen elimination. FXIIa activates the kallikrein–kinin system. BK, formed during this reaction, mediates various inflammatory effects, including vasodilation, pain, leukocyte chemotaxis, and adhesion [149,150,151]. Primed neutrophils can bind and activate plasma FXII [100,294]. HK and PK also stick to proteoglycans on the neutrophil’s surface [214,295,296] and modulate the release of BK [297]. FXIIa, generated by this system, in turn, induces neutrophil activation and degranulation [148]. FXIIa and HK compete for binding to uPAR domain II [214], but have an opposite effect on cells: FXIIa induces and HK blocks intracellular signal transduction [215].

FXIIa interacts with the classical complement pathway followed by a generation of C3a and C5a anaphylatoxins. C1INH, which controls the activity of the complement system, is the main inhibitor of the contact system with FXIIa and PK as targets [298] (Figure 3, Table 2).

Thrombin is also part of the complex coagulo-complement network. In high concentrations, thrombin is able to cleave C3a and C5a complement components [159]. Some authors consider these reactions as the fourth complement cascade activation [160] (Figure 3, Table 2).

Thrombin also has a proteolytic effect on the 15-AA fragment of chemerin (YFPGQFAFSKALPR): chemerin-15 (Figure 3, Table 2). If chemerin-15 is practically inert, both chemerin-14 and chemerin-10 fragments, cleaved by thrombin, have a chemoattractant effect on macrophages, dendritic cells, and natural killer cells, expressing the chemerin receptor CMKLR1 [155,156,157,158,299]. Chemerin increases the expression of inflammatory mediators IL-6, TNFα, and the C-reactive protein on endothelial cells, and their adhesiveness to leukocytes [300,301,302,303,304]. Chemerin enhances mitochondrial ROS generation [303] and suppresses nitric oxide-mediated vasodilatation [305,306,307].

Another important mechanism for triggering inflammation is thrombin-induced IL-1α production. IL-1α is translated as a pro-form with minor bioactivity (pro-IL-1α). A number of proteases can cleave pro-IL-1α into a mature form of the cytokine with several-fold higher bioactivity (Figure 3, Table 2). This usually proceeds intracellularly, with inflammasome assembly and caspase activation, induced under the influence of oxidative stress, ischemia reperfusion, and radiation. However, thrombin also can cleave pro-ILα released from necrotic cells. The physiological significance of the mechanism for the development of an inflammatory reaction was confirmed in studies on transgenic mice [308] and atherogenesis [193].

In addition, the ability of thrombin to enzymatically cleave IL-33 was observed [130] (Figure 3, Table 2). IL-33 is a tissue-derived nuclear cytokine produced mainly by endothelial, epithelial, epidermal, and fibroblast-like cells, and myofibroblasts, but not by hematopoietic cells. IL-33, accumulated from damaged cells, acts as alarmin [309]. It was found that LMWH, suppressing the cleavage of IL-33, downregulates type 2 inflammation induced by both papain and fungi in mice [130]. Bivalirudin suppressed type 2 immune reactions in models of lung inflammation caused by house dust mites and ovalbumin. The levels of the thrombin–antithrombin complex in the blood plasma of patients with bronchial asthma positively correlated with the number and function of Innate Lymphoid Cells 2 (ILC2s) responding to IL-33 [130].

### 5.2. Coagulation Factors and Vascular Inflammation

The location between tissues and blood determines the equal involvement of the vascular endothelium in the processes of coagulation and inflammation. At the site of inflammation, the endothelium slows down blood flow, attracts leukocytes, and induces microthrombus formation to limit pathogen dissemination [310] (Figure 3, Table 2).

FXIIa, through uPAR, directly contributes to the formation of a pro-inflammatory phenotype, the mitogenic/angiogenic activity of endothelial cells, and increases vascular permeability due to BK generation. The binding of FXIIa to uPAR in endothelial cells depends on Zn^2+^ ions released from platelets [46,215,311,312]. The assembly of the protein complexes of the contact system on the surface of endothelial cells stimulates the kallikrein–kinin system’s activation, controlled by FXIIa [313]. It is assumed that endothelial cells, like neutrophils, can produce FXII [314]. Kinins, through the activation of GPCRs and bradikinin receptors, B1R and B2R [315,316], cause vasodilation and increase vascular permeability, edema, pain, as well as ROS generation and endothelial inflammation [31,166,167]. B2R constitutively expresses in various tissues and upregulates with injuries or infectious diseases [317,318,319,320]. On the other hand, B1R is almost undetectable under physiological conditions, but increases after the activation of NF-κB, c-Jun, and the p38 signaling cascade in endothelial cells [321]. The expression of B1R enhances under the influence of IL-1β, TNF, and IFNγ cytokines [322,323] in pathological conditions, such as tissue or vascular damage, ischemia, diabetes mellitus, as well as infection [318,320,322,324,325]. Ca^2+^/calmodulin, ERK, MAPK, and PhosphoInositide 3-Kinase (PI3K)/Akt intracellular pathways, triggered by B1R and B2R, increase nitric oxide synthase expression, nitric oxide [169,170], and VEGF production [171]. Bradikinin receptor-induced ERK and NF-κB activation also result in the expression of cyclooxygenase 2 and the production of prostaglandins and prostacyclin [172]. The congenital deficiency of C1INH, the main inhibitor of FXIIa and pKa, observed in hereditary angioedemas of types I and II, is the finest demonstration of FXII involvement in pathological vascular reactions [326].

FXI is a component of the contact system and a downstream target of FXIIa. Thrombin-mediated FXI activation, as a positive feedback mechanism [327,328,329,330], supports thrombin production and prevents premature clot lysis [331,332] (Figure 1). In sepsis models, it was found that (FXI−/−) mice with an FXI deficiency have a weakened cytokine storm during an acute phase of infection and a higher survival rate [101]. Studies on a hyperlipidemia model of non-human primates have shown that FXI activation contributes to the development of a pro-inflammatory endothelial phenotype with platelet activation and subsequent endothelial dysfunction [333].

A variety of reactions triggered by thrombin on the endothelium support inflammatory processes in atherosclerosis, massive thrombosis, vasculitis [173,334,335,336], and autoimmune [50] and allergic diseases [65,66]. Thrombin affects endothelial cells via PARs 1, 2, and 4 [337]. Thrombin-mediated changes in the endothelial phenotype, such as an increase in cytokine (IL-6 [173], MIF [175], IL-8 [174], fractalkin, MCP-1 production [176], and expression of adhesion molecules (vascular cell adhesion molecule 1 (VCAM-1), ICAM-1, and E- and P-selectins) [177,178,179], regulate the formation of leukocyte infiltrates [338]. Moreover, thrombin can stimulate the synthesis and release of a wide range of biologically active substances, including prostacyclin [180,181], PAF [182], endothelin [183], the von Willebrand factor [184], the Plasminogen Activator, and its inhibitor, regulating vascular permeability, tone, and angiogenesis [230,339].

Fibrin is a ligand of various adhesion molecules that determines its role in regulating intercellular interactions during leukocyte transendothelial migration [238,264,340]. Fibrin or its degradation products serve as molecular bridges connecting β2-integrins on leukocytes and VE-cadherins on endothelial cells [186]. This is confirmed by in vitro studies demonstrating that the fibrin N-terminal disulphide knot promotes aXβ2 integrins and VE-cadherin-mediated leukocyte migration. Fibrinogen enhances the adhesion of leukocytes to the endothelium through ICAM-1 [187]. This mechanism is amplified by a fibrinogen–VE-cadherin-induced ICAM-1 expression. The open sequence of fibrinogen Bβ15–42 is necessary for VE-cadherin binding, since FpB peptide release prevents fibrinogen–VE-cadherin binding and ICAM-1 expression [341]. Another potential mechanism that promotes leukocyte transmigration is that fibrin (but not fibrinogen) or its breakdown products activate the endothelium through a very low-density lipoprotein receptor [342].

According to the investigation of Prasad et al., mice, expressing a mutant form of fibrinogen, with retarded fibrin polymerization, demonstrate a weakened antimicrobial response [343]. This shows that fibrin, rather than fibrinogen, is crucial for antimicrobial mechanisms.

### 5.3. Adaptive Immunity

The key event triggering adaptive reactions is the formation of a pool of mature dendritic cells that deliver antigens from a site of injury to regional lymphoid nodes and present them to naive T cells. As a rule, PRR ligands (PAMPs and DAMPs) act as adjuvants that induce dendritic cell maturation. Thrombin, formed during an infection and/or hemostasis, can also act as an adjuvant. In the study by Yanagita et al., it was found that thrombin-threated dendritic cells increase the expression of the Human Leukocyte Antigen–DR isotype (HLA-DR), CD86, the ability to produce MCP-1 and IL-12, and stimulate the proliferation of allogeneic T lymphocytes [198]. Under the influence of thrombin, mature dendritic cells produce CCL18, a chemoattractant that promotes dendritic cells and T-lymphocyte colocalization [199]. Thrombin, due to its property to cleave osteopontin (Thr-OPN), can also attract dendritic cells at the site of injury [344]. In physiological concentrations, thrombin significantly enhances T-cell proliferation in response to mitogens, superantigens, alloantigens, and stimulation with anti-CD3 antibodies [345]. Finally, it has been shown that thrombin plays the key role stimulating the alloimmune reactions of T cells in ischemic reperfusion tissue injury after transplantation [346] (Figure 3, Table 2).

In the case of physiological hemostasis, the activated products of the coagulation system can contribute to the formation of definite types of immune reactions aimed at regeneration, which correspond to type 2 inflammation and a Th2 adaptive immune response. It is noteworthy that many allergens, as well as components of the coagulation cascade, show serine protease activity and often trigger a type 2 inflammatory response [347]. Perhaps allergens mimic the activity of the components of the coagulation cascade and the immune system initiates a regenerative type of inflammation as if a tissue injury occurs. Unlike coagulation cascade activation, the wave of which eventually fades, allergic reactions tend to intensify. However, the data of the literature in this field appear controversial. Thrombin is known to induce the anti-inflammatory M2-like phenotype of macrophages, in which the profile of the expressed genes (CCL22, CD36, and MMP9) corresponds to an intermediate state between M2a and M2c [190] (Figure 3, Table 2). Thrombin-treated macrophages enhance OxLDL phagocytosis, and the medium, conditioned with these cells, increases endothelial cell proliferation [190]. The recently discovered ability of thrombin to cleave mature IL-33 also speaks in favor of the protease to participate in triggering type 2 inflammation [130] (Figure 3, Table 2).

At the same time, other studies found that thrombin directs the polarization of macrophages, derived from murine bone marrow monocytes, into an M1-like inflammatory phenotype, characterized by iNOS, Fpr2, ectoenzyme CD38, and proinflammatory cytokines IL-6, TNFα, and IP-10 [190] (Figure 3, Table 2).

It was also found that FXIIa promotes the polarization of naive T helper cells (Th0) into Th17 cells, regulating a neutrophil type of inflammation, one of the most harmful in terms of tissue damage [53,214] (Figure 3, Table 2).

The discrepancy between the results obtained by different researchers may be related to various experimental models. In addition, immune cell differentiation can be easily affected by microenvironment conditions, cytokines, and other inflammatory mediators. Overall, the scarce data do not allow us to draw an unambiguous conclusion on the type of adaptive immune reactions that evolve under the influence of coagulation factors.

## 6. Conclusions

Currently, a sufficient amount of data demonstrate the activated coagulation factors to be an endogenous «danger» signal for the immune system. The coagulation system has all the properties inherent in DAMPs: launching the production of inflammatory mediators, the activation of the endothelium, and increasing vascular permeability, the activation of immune system cells, and attracting them to the source of inflammation. The coagulation system can recognize signs of pathogenicity, as well as activate innate and adaptive immunity, and even probably affect a type of inflammation and adaptive immune response. Altogether, it allows us to consider the coagulation cascade proteins as a separate, long-known group of DAMPs. Procoagulant factors support local and systemic inflammatory reactions, which leads to an increase in the severity of the disease, initiation and maintenance of multiple organ failure, and the spread of the pathological process. The development of new therapies aimed at suppressing coagulation factors in inflammatory diseases will be able to solve various difficulties in treating patients. However, it is necessary to take into account the individual characteristics of the patient, the presence of problematical indications, and the course of the underlying disease when deciding on the suppression of coagulation. Further studies of the coagulation system will certainly expand our understanding of the physiology and pathophysiology of inflammation.

## Figures and Tables

**Table 1:** Examples of coagulation and inflammation crosstalk in various pathologies.

Disease	Coagulation Factor	Mechanism of Action	Clinical Significance	Ref.
Pneumonia	FXII, FXI, TF, PF, D-dimer, fibrinogen, and thrombin	Blood clot formation, initiating local or systemic vascular endothelial damage, activation of neutrophils, and NET formation	Worsening of respiratory symptoms, increased risk of thromboembolic events, maintenance of febrile syndrome, initiation of ARDS, and increase in disease severity and mortality	[37]
Sepsis	TF, FXI, and FXII	Polymicrobial infection maintenance, and monocyte and neutrophil activation	Initiation of disseminated intravascular coagulation syndrome, septic shock, and multiple organ failure	[12,38]
HIV	PAI-1 and TF	Activation of neutrophils and NET formation	Viral load maintenance	[39,40,41,42,43,44]
Metabolic disorders	FXII, von Willebrand factor, tPA, PAI-1, FVII and FVIII, and thrombin	Activation of NF-κB and caspase-3 in leukocytes, plaque progression, and leukocyte recruitment	Development of glucose intolerance, cardiac fibrosis, and inflammation	[45,46]
Chronic autoimmune urticaria	Prothrombin	T-cell activation and chemotaxis	Increase in disease severity	[47,48,49,50]
Multiple sclerosis	FXII and FXI	Stimulation of Th17 lymphocyte polarization	Relapse initiating	[51,52,53,54]
Inflammatory arthropathies	Fibrin, D-dimer, prothrombin, and PAR-1	Stimulation of cell hyperproliferation and inflammation	Increase in disease severity	[18,55,56,57,58,59,60,61,62,63]
Antiphospholipid syndrome	TF	Endotheliocyte and neutrophil activation	Induction of occlusive vasculopathy and organ failure	[64]
Allergic diseases	Thrombin, PAR-1, and FXII	Stimulation of epithelium permeability and eosinophil migration into the respiratory tract, hypertrophy of goblet cells, and mucus secretion	Increase in bronchial tone and severity of anaphylaxis	[65,66]
Cancer	Thrombin, PAR-1, and fibrin	Inhibition of natural killer cell activity, induction of differentiation of myeloid suppressor cells, M2-like macrophages, and Tregs	Increase in disease severity and metastasis	[67,68,69,70,71,72,73,74,75]
Diabetic kidney disease	FXII	Induction of oxidative stress and tubular cell injury	Initiation of organ dysfunction	[76]
Sickle cell disease	FXII and thrombin	Enhancement of TNFα-induced inflammation and neutrophil adhesion	Initiation of venous thrombosis and microvascular stasis	[77]

Abbreviations: ARDS, acute respiratory distress syndrome; HIV, human immunodeficiency virus; FIIa, activated Factor II; FXIIa, activated Factor XII; FVII, Factor VII; FVIII, Factor VIII; FXI, Factor XI; NET, neutrophil extracellular trap; NF-κB, nuclear factor κB; PAI-1, plasminogen activator inhibitor-1; PAR-1, protease-activated receptor 1; PF, platelet factor; TF, tissue factor; TNFα, tumor necrosis factor alpha; tPA, tissue plasminogen activator; Treg, regulatory T cell.

**Table 2:** Impact of coagulation factors on innate immunity cells.

Cell Type	Coagulation Factor	Effect	References
Neutrophil	FXIIa	Degranulation	[148]
		Bradykinin generation	[149,150,151]
		Increase in intracellular calcium and NET release	[152]
		Increase in TLR-mediated activation	[153].
		Upregulation of αMβ2 integrin	[100]
	FIIa	Chemoattraction	[154]
		Chemerin-14 and chemerin-10 fragment generation	[155,156,157,158]
		C3 and C5 complement component production	[159,160]
	Fibrinogen	Leukocyte adhesion and transendothelial migration	[161]
		Cytokine/chemokine production	[162,163,164],
		ROS production	[165].
Endothelial cell	FXIIa	Bradykinin-mediated vasodilation	[166,167,168]
		Edema	[166,167,168]
		Vascular permeability	[166,167,168]
		ROS generation	[166,167,168]
		Nitric oxide production	[169,170]
		VEGF production	[171]
		Prostaglandin/prostacyclin production	[172]
	FIIa	Production of IL-6	[173]
		IL-8 production	[174]
		MIF production	[175]
		Fractalkin (CX3CL1) production	[176]
		MCP-1 production	[176]
		VCAM-1 and ICAM-1	[177,178]
		E- and P-selectin expression	[177,179]
		Prostacyclin production	[180,181]
		Platelet activating factor expression	[182]
		Endothelin expression	[183]
		von Willebrand factor expression	[184]
	Fibrinogen	Cytokine/chemokine production	[185]
		Leukocyte β2-integrin and VE-cadherin interaction	[186]
		ICAM-1 upregulation	[187]
		ICAM-1-mediated leukocyte endothelium adhesion	[187]
Monocyte/ macrophage	FXIIa	TNFα, IL-1β, IL-12, and IL-6 production	[188]
		LPS-mediated IL-1α production	[189]
	FIIa	Macrophage polarization	[190]
		IL-1α production	[191,192,193]
		IL-33 production	[130]
	FVIIIa	Fcγ and complement receptor-mediated phagocytosis	[194]
Mast cell	FIIa	Degranulation	[195]
		VEGF, TNFα, IL-6, CCL-2, CXCL-1, and CXCL-5 production	[47,195]
	Fibrinogen	IL-13-mediated PD-L2+ dendritic cell differentiation and Th2 immune response	[196]
Dendritic cell	FXIIa	Inflammatory cytokine production	[53]
		Differentiation of antigen-specific IFNy producing CD4+ T cells	[197]
	FIIa	HLA-DR and CD86 expression	[198]
		MCP-1 and IL-12 production	[198]
		Allogenic T-cell proliferation	[198]
		CCL18-mediated immature DC and TC co-localization	[199]
	FVIIIa	CCL19-mediated DC lymph node migration	[200]
T cell	FIIa	Th 17 polarization	[53]

Abbreviations: DC, dendritic cell; ICAM-1, intercellular adhesion molecule 1; IFNγ, interferon gamma; HLA-DR, HLA-DR; IL, interleukin; LPS, lipopolysaccharide; MCP-1, monocyte chemoattractant protein-1; MIF, migration inhibition factor; NETs, neutrophil extracellular traps; PD-L2, programmed cell death 1 ligand 2; ROS, reactive oxygen species; Th, T helper cell; TLR, Toll-like receptor; TNFα, tumor necrosis factor alpha; VCAM-1, vascular cell adhesion molecule 1; VEGF, vascular endothelial growth factor; VE-cadherin, Vascular endothelial cadherin.

## Data Availability

Not applicable.
